# Correlation and Prognostic Significance of the Hemoglobin, Albumin, Lymphocyte, and Platelet (HALP) Score in Acute Ischemic Stroke Survivors

**DOI:** 10.7759/cureus.102355

**Published:** 2026-01-26

**Authors:** Neelam Javed, Hina Hanif, Aruljanani Sachidanantham, Tehseen Tanveer, Maria Khan, Summaya Mehboob, Muhammad Zulfiqah Sadikan, Sana Maryam

**Affiliations:** 1 Internal Medicine, Liaquat College of Medicine and Dentistry, Karachi, PAK; 2 Internal Medicine, Sharif Medical City Hospital, Lahore, PAK; 3 Internal Medicine, SP Medical Center Pvt Ltd, Chennai, IND; 4 General Medicine, KRL Hospital, Islamabad, PAK; 5 General Medicine, Pakistan Institute of Medical Sciences (PIMS), Islamabad, PAK; 6 Medicine, Glangwili General Hospital, Carmarthen, GBR; 7 Internal Medicine, Sandeman Provincial Hospital, Quetta, PAK; 8 Pharmacy and Health Sciences, Universiti Kuala Lumpur Royal College of Medicine Perak, Ipoh, MYS; 9 Pharmacology, Faculty of Pharmacy, Islamia University of Bahawalpur, Bahawalpur, PAK

**Keywords:** acute ischemic stroke, inflammation, outcome, patients, prognosis

## Abstract

Background: Acute ischemic stroke (AIS) remains a leading cause of death and long-term disability worldwide. Early identification of high-risk patients is crucial for improving outcomes.

Objective: This study aimed to determine the correlation and prognostic significance of the Hemoglobin, Albumin, Lymphocyte, and Platelet (HALP) score in patients with AIS.

Methodology: This cross-sectional analytical study was conducted at the Liaquat College of Medicine and Dentistry, Karachi, Pakistan, from June 2024 to September 2025. A total of 355 patients diagnosed with AIS were enrolled using non-probability consecutive sampling. Hemoglobin, serum albumin, lymphocyte count, and platelet count were obtained within 24 hours of admission, and the HALP score was calculated as (hemoglobin × albumin × lymphocyte count)/platelet count.

Results: The mean age of participants was 60.8 ± 11.9 years, with 58.6% males. The mean HALP score was 32.6 ± 16.4. Patients with favorable outcomes (modified Rankin Scale (mRS): 0-2) had significantly higher HALP scores (39.8 ± 14.1) than those with poor outcomes (mRS: 3-6; 22.9 ± 12.6; p < 0.001). HALP was negatively correlated with the National Institutes of Health Stroke Scale (NIHSS) (r = −0.62; p < 0.001) and positively associated with functional independence (r = 0.55; p < 0.001). In multivariate logistic regression, HALP independently predicted poor functional outcome (adjusted odds ratio (OR) = 0.91; 95% CI: 0.87-0.95; p < 0.001) and in-hospital mortality (adjusted OR = 0.89; 95% CI: 0.82-0.96; p = 0.002).

Conclusion: A lower HALP score is strongly associated with greater stroke severity, worse functional recovery, and higher mortality in patients with AIS.

## Introduction

Acute ischemic stroke (AIS) remains a major global health challenge and continues to rank among the leading causes of death and long-term neurological disability worldwide [1]. Its incidence is rising, largely driven by an aging population and the increasing prevalence of modifiable risk factors such as hypertension, diabetes, dyslipidemia, and smoking [2]. Following an ischemic event, early and accurate prognostication is essential, as it guides treatment decisions, rehabilitation planning, and expectations for functional recovery [3]. Although several clinical and imaging-based scoring systems are available, the need persists for biochemical markers that capture the combined inflammatory and nutritional status of the patient. Inflammation plays a central role in the evolution of ischemic brain injury, contributing to neuronal damage, secondary ischemic cascades, and delayed recovery [4]. Effective recovery requires controlling microglial activation, limiting inflammatory cell infiltration, and resolving cytokine-mediated injury. Nutritional status is equally important; adequate nutrition supports neuroplasticity and repair, whereas malnutrition weakens immune competence and slows functional recovery [5]. Integrating inflammatory and nutritional indicators may therefore offer a more comprehensive reflection of the patient’s physiological reserve and recovery potential [6].

Each component of the Hemoglobin, Albumin, Lymphocyte, and Platelet (HALP) score contributes distinct clinical insight: hemoglobin reflects oxygen-carrying capacity, albumin indicates nutritional and metabolic status, lymphocytes reflect immune competence, and platelets represent coagulation activity. Individually, these markers have been linked to outcomes across a range of vascular and systemic diseases [7], but their combined use provides a more synergistic representation of the complex interplay between inflammation, nutrition, and hemostasis. More recent studies have shown that lower HALP scores in patients with various cardiovascular and metabolic conditions are associated with higher mortality, greater complications, and recovery stagnation [8]. Strokes have overlapping mechanisms with these conditions, so the HALP score potentially may be a good prognostic tool in patients with AIS. Some of the individual HALP components already have prognostic value in stroke [9]. For instance, anemia worsens cerebral hypoxia and enlarges the infarct, and also causes hypoalbuminemia, which leads to higher mortality and poor functional outcomes due to its effect on vascular permeability and oxidative stress [10]. Lymphopenia indicates immune vulnerability and increases the likelihood of acquiring infections after a stroke. In contrast, thrombocytosis is the abnormal increase in thrombocyte levels in blood and can precipitate thrombosis and trigger secondary ischemic events. When all these factors are put together, the HALP score can give one simple, combined systemic measure of systemic vulnerability [11]. HALP is simple and inexpensive to use, as one score can be derived from multiple blood tests. This makes HALP scores viable for countries and areas with limited resources [12]. This study aimed to determine the correlation and prognostic significance of the HALP score in patients with AIS.

## Materials and methods

This cross-sectional analytical study was conducted at the Liaquat College of Medicine and Dentistry, Karachi, Pakistan, from June 2024 to September 2025. A total of 355 patients diagnosed with AIS were enrolled in the study. Participants were selected using a non-probability consecutive sampling technique. The study included adult patients aged 18 years and above who had a confirmed diagnosis of AIS based on CT or MRI findings and who presented within 72 hours of symptom onset. Patients were excluded if they had hemorrhagic stroke or transient ischemic attacks, a known history of malignancy, chronic inflammatory or autoimmune disorders, or significant hepatic or renal dysfunction. Individuals with incomplete clinical or laboratory records were also excluded to ensure data accuracy and reliability.

Data collection

Following ethical approval, patient data were collected from both hospital medical records and direct clinical assessment. Demographic details (age, sex), medical history (hypertension, diabetes mellitus, dyslipidemia, and smoking status), and clinical parameters such as the time of stroke onset, Glasgow Coma Scale (GCS), and the National Institutes of Health Stroke Scale (NIHSS) score at admission were recorded. Laboratory investigations, including hemoglobin (g/dL), serum albumin (g/L), total lymphocyte count (×10⁹/L), and platelet count (×10⁹/L), were obtained from blood samples collected within 24 hours of hospital admission. Using these parameters, the HALP score was calculated with the following formula: HALP = (hemoglobin (g/dL) × albumin (g/dL) × lymphocyte count (×10⁹/L))/platelet count (×10⁹/L).

The functional outcome of each patient was assessed using the modified Rankin Scale (mRS) at the time of hospital discharge and again at the three-month follow-up. Based on the mRS scores, patients were categorized into two groups: favorable outcome group (mRS: 0-2) and poor outcome group (mRS: 3-6). In-hospital mortality was also recorded as a secondary outcome measure.

Statistical analysis

Data were analyzed using IBM SPSS Statistics for Windows, Version 26 (Released 2018; IBM Corp., Armonk, New York, United States). Continuous variables such as age, hemoglobin, albumin, lymphocyte count, platelet count, and HALP score were expressed as mean ± standard deviation (SD). Categorical variables, including gender, comorbidities, and outcome groups, were summarized as frequencies and percentages. The correlation between the HALP score and stroke severity (NIHSS score) was analyzed using Pearson’s correlation coefficient. A p-value of <0.05 was considered statistically significant.

## Results

Data were collected from 355 patients, and the mean age of all patients was 60.8 ± 11.9 years, with those achieving a favorable outcome (mRS: 0-2) being significantly younger (58.1 ± 11.2 years) than those with poor outcomes (64.7 ± 10.9 years; p < 0.001). Of the total population, 208 (58.6%) were males, with no significant gender difference between groups (59.4% vs. 57.3%; p = 0.71). Hypertension was the most prevalent comorbidity, affecting 238 (67.0%) patients, and was markedly higher in the poor outcome group (77.6%) compared to the favorable outcome group (59.9%). Similarly, diabetes mellitus was more common among patients with poor outcomes (76 patients; 53.1%) than among those with good outcomes (78 patients; 36.8%; p = 0.003). Dyslipidemia was seen in 111 (31.3%) patients, again higher in poor outcomes (37.8%) than in favorable ones (26.9%). Interestingly, smoking was reported more frequently among patients with favorable outcomes (32.5%) than poor ones (21.7%; p = 0.031). The mean NIHSS score at admission was 10.7 ± 4.8, significantly higher in poor outcome patients (14.1 ± 4.3) compared to the favorable ones (8.4 ± 3.9) (Table 1).

**Table 1:** Baseline characteristics of the study population (n = 355) NIHSS: National Institutes of Health Stroke Scale: HALP score: Hemoglobin, Albumin, Lymphocyte, and Platelet score; mRS: modified Rankin Scale The primary outcome was functional status at 90 days, assessed using the mRS. Outcomes were dichotomized into favorable (mRS: 0-2) and poor (mRS: 3-6). The 90-day time point is internationally recognized as the standard endpoint in stroke research because it reflects stable neurological recovery, reduces variability related to acute-phase care, and aligns with major clinical trials and guideline recommendations

Variable	Overall (n = 355)	Favorable outcome (mRS: 0-2; n = 212)	Poor outcome (mRS: 3-6; n = 143)
Age (years), mean ± SD	60.8 ± 11.9	58.1 ± 11.2	64.7 ± 10.9
Male, n (%)	208 (58.6%)	126 (59.4%)	82 (57.3%)
Hypertension, n (%)	238 (67.0%)	127 (59.9%)	111 (77.6%)
Diabetes mellitus, n (%)	154 (43.4%)	78 (36.8%)	76 (53.1%)
Dyslipidemia, n (%)	111 (31.3%)	57 (26.9%)	54 (37.8%)
Smoking, n (%)	100 (28.2%)	69 (32.5%)	31 (21.7%)
NIHSS at admission, mean ± SD	10.7 ± 4.8	8.4 ± 3.9	14.1 ± 4.3
HALP score, mean ± SD	32.6 ± 16.4	39.8 ± 14.1	22.9 ± 12.6

The correlation analysis revealed a strong negative correlation between HALP and NIHSS at admission (r = −0.62; p < 0.001), indicating that lower HALP values correspond to more severe neurological impairment. Likewise, the correlation between HALP and mRS at three months (r = −0.58; p < 0.001) confirmed that patients with lower HALP scores experienced poorer functional outcomes. The HALP score also showed a moderate inverse relationship with age (r = −0.31; p = 0.002), suggesting that older individuals tend to have lower HALP values. On the other hand, HALP was positively correlated with serum albumin (r = +0.64; p < 0.001) and hemoglobin (r = +0.47; p < 0.001) (Table 2).

**Table 2:** Correlation of HALP score with clinical and laboratory parameters NIHSS: National Institutes of Health Stroke Scale: HALP score: Hemoglobin, Albumin, Lymphocyte, and Platelet score

Parameter	Correlation coefficient (r)	p-value
NIHSS at admission	-0.62	<0.001
Modified Rankin Scale (three months)	-0.58	<0.001
Age (years)	-0.31	0.002
Serum albumin	+0.64	<0.001
Hemoglobin	+0.47	<0.001

For every one-year increase in age, the odds of poor recovery increased by 4% (adjusted odds ratio (OR) = 1.04; 95% CI: 1.01-1.07; p = 0.013). Diabetic patients were 1.58 times more likely to experience poor outcomes than non-diabetics (95% CI: 1.03-2.43; p = 0.036). Stroke severity at admission, reflected by NIHSS, was a strong determinant of prognosis; each one-point rise in NIHSS increased the odds of poor outcome by 29% (adjusted OR = 1.29; 95% CI: 1.17-1.43; p < 0.001). Importantly, HALP score remained a robust protective factor even after adjusting for confounders; each unit increase in HALP reduced the risk of poor outcome by 9% (adjusted OR = 0.91; 95% CI: 0.87-0.95; p < 0.001) (Table 3).

**Table 3:** Logistic regression analysis for the predictors of poor functional outcome (mRS: 3-6) NIHSS: National Institutes of Health Stroke Scale: HALP score: Hemoglobin, Albumin, Lymphocyte, and Platelet score; mRS: modified Rankin Scale

Variable	Adjusted odds ratio (OR)	95% confidence interval (CI)	p-value
Age (years)	1.04	1.01-1.07	0.013
Diabetes mellitus	1.58	1.03-2.43	0.036
NIHSS at admission	1.29	1.17-1.43	<0.001
HALP score	0.91	0.87-0.95	<0.001

The mean age declined progressively from 63.9 ± 10.8 years in Tertile 1 to 57.9 ± 12.1 years in Tertile 3 (p = 0.002), suggesting that higher HALP values are more common in younger patients. The mean NIHSS score significantly decreased across tertiles, from 13.8 ± 4.2 in the lowest HALP group to 8.0 ± 3.5 in the highest (p < 0.001), indicating that higher HALP scores are associated with milder strokes. Similarly, the mean three-month mRS score improved steadily, from 4.2 ± 1.3 in Tertile 1 to 1.9 ± 0.9 in Tertile 3 (p < 0.001). The rate of favorable outcomes (mRS: 0-2) increased dramatically from 28.8% in the lowest tertile to 88.0% in the highest (p < 0.001), while in-hospital mortality declined from 11.9% to 2.6% (p = 0.009) (Table 4). 

**Table 4:** Association of the HALP score tertiles with stroke severity and clinical outcomes (n = 355) NIHSS: National Institutes of Health Stroke Scale: HALP score: Hemoglobin, Albumin, Lymphocyte, and Platelet score; mRS: modified Rankin Scale

HALP score tertiles	Tertile 1 (<25.0)	Tertile 2 (25.0-40.0)	Tertile 3 (>40.0)	p-value
No. of patients, n (%)	118 (33.2%)	120 (33.8%)	117 (33.0%)	-
Age (years), mean ± SD	63.9 ± 10.8	60.7 ± 11.5	57.9 ± 12.1	0.002
Male, n (%)	65 (55.1%)	73 (60.8%)	70 (59.8%)	0.64
NIHSS score at admission, mean ± SD	13.8 ± 4.2	10.4 ± 3.8	8.0 ± 3.5	<0.001
Modified Rankin Scale (mRS three months), mean ± SD	4.2 ± 1.3	2.8 ± 1.1	1.9 ± 0.9	<0.001
Favorable outcome (mRS: 0-2), n (%)	34 (28.8%)	75 (62.5%)	103 (88.0%)	<0.001
In-hospital mortality, n (%)	14 (11.9%)	9 (7.5%)	3 (2.6%)	0.009

## Discussion

This study examined the correlation and prognostic value of the HALP score in patients with AIS. Among the 355 enrolled patients, the findings demonstrated that lower HALP scores were consistently associated with greater stroke severity, worse neurological outcomes, and higher in-hospital mortality. Because the HALP score integrates nutritional, inflammatory, and hematologic status, it serves as a simple yet clinically meaningful indicator of systemic resilience in AIS patients. The strong negative correlation between HALP and NIHSS confirms that patients with lower HALP values tend to present with more severe neurological deficits. This relationship is biologically plausible: systemic inflammation and malnutrition both impair the body’s ability to tolerate and recover from ischemic injury. Excessive inflammatory activity following cerebral ischemia accelerates oxidative stress, disrupts vascular repair, and expands the zone of neuronal injury [13]. Lower lymphocyte counts and hypoalbuminemia, reflected in reduced HALP scores, are well-recognized markers of impaired immune competence, poor nutritional status, and heightened systemic stress, all of which are linked to unfavorable neurological recovery.

The marked difference in mean HALP scores between outcome groups in this study (39.8 vs. 22.9; p < 0.001) highlights its strong discriminatory ability. Prior research has shown the utility of the HALP score in predicting complications and survival in oncology and cardiology cohorts, and emerging evidence suggests prognostic relevance in broader ischemic and inflammatory conditions [14]. Individually, anemia, hypoalbuminemia, and lymphopenia have each been associated with post-stroke mortality and long-term disability; our findings reinforce that their combined effect captured through the HALP score offers superior prognostic strength compared with evaluating each parameter in isolation. Multivariate logistic regression further confirmed HALP as an independent predictor of both poor functional outcomes and in-hospital mortality, even after adjustment for key confounders such as age, diabetes, and NIHSS score [15]. This highlights that the HALP score captures a unique pathophysiological profile, integrating inflammation, nutritional reserve, oxygen-carrying capacity, and immune competence factors that conventional stroke severity scales do not account for [16]. Overall, the findings reinforce HALP as a practical, low-cost, and biologically meaningful prognostic marker that can enhance risk stratification and clinical decision-making in AIS.

HALP and mortality correlation

Patients in the lowest tertile of HALP overall score had grossly higher in-hospital death rates (11.9%) than those in the highest tertile (2.6%). This supports its prognostic credibility. Albumin and hemoglobin contribute to the HALP score and explain some differences in outcome because HALP incorporates score features of outcome relevant to the score's physiology. Having neuroprotective qualities and defending against inflammation as well as regulating intravascular oncotic pressure and the endothelium, serum albumin prevents functional decline and is neuroprotective [17]. Hemoglobin's role in oxygen transport is critical, and deficiency results in oxygen deprivation to the brain, contributing to hypoxia, ischemic brain lesions, and injury.

Lymphocytes and Inflammation of the Brain in Stroke

Thrombosis and ensuing vascular events of the brain are associated with platelets. HALP is a composite of features that measure score physiology. HALP is a composite of features that measure score physiology and is especially important in a situation with multiple systems involved in the disease process, as is the case with AIS [18].

There are clinical implications to the findings. Since all the components are obtained from simple routine laboratory tests, the HALP score is beneficial in clinical practice at no extra cost. This holds especially true in the developing world, such as Pakistan, where routine advanced imaging and stroke biomarkers are not easily available. The study has several important limitations. First, as an observational cohort design, it cannot establish causality, and residual confounding may persist despite multivariable adjustments. Treatment-related variables such as thrombolysis, thrombectomy, antiplatelet timing, and rehabilitation intensity were not captured, which may influence outcomes and HALP associations. Laboratory parameters used for HALP calculation were obtained only once at admission, limiting insight into dynamic changes over time. Additionally, single-center data and a geographically restricted population may reduce generalizability. The study also did not account for nutritional assessments, inflammatory biomarkers beyond HALP components, or stroke subtypes, all of which could affect prognostic interpretation. Finally, although smoking appeared associated with better outcomes, this likely reflects survivor or selection bias rather than a true protective effect, underscoring the potential for unmeasured confounders.

## Conclusions

It is concluded that the HALP score serves as a valuable prognostic indicator in patients with AIS. Lower HALP scores were consistently associated with greater stroke severity, poorer functional recovery, and higher in-hospital mortality. Because the score integrates hematologic, nutritional, and inflammatory parameters, it provides insight into physiological domains not captured by conventional stroke severity tools. Given its simplicity, low cost, and availability from routine laboratory tests, the HALP score may be incorporated into early risk stratification to guide clinical decision-making, identify high-risk patients, and optimize post-stroke management. A major novelty of this study is that it provides the first evidence from a Pakistani AIS population demonstrating that the HALP score is a powerful and independent predictor of stroke severity, functional outcome, and in-hospital mortality. Unlike traditional clinical scales such as the NIHSS, the HALP score uniquely integrates inflammatory, nutritional, hematologic, and immune parameters into a single biomarker, capturing a distinct pathophysiological dimension that routine clinical assessments often overlook.
